# Towards Clinical Molecular Diagnosis of Inherited Cardiac Conditions: A Comparison of Bench-Top Genome DNA Sequencers

**DOI:** 10.1371/journal.pone.0067744

**Published:** 2013-07-04

**Authors:** Xinzhong Li, Andrew J. Buckton, Samuel L. Wilkinson, Shibu John, Roddy Walsh, Tomas Novotny, Iveta Valaskova, Manu Gupta, Laurence Game, Paul J R. Barton, Stuart A. Cook, James S. Ware

**Affiliations:** 1 National Heart and Lung Institute, Imperial College, London, United Kingdom; 2 NIHR Biomedical Research Unit in Cardiovascular Disease, Royal Brompton and Harefield NHS Foundation Trust and Imperial College London, London, United Kingdom; 3 Department of Internal Medicine and Cardiology, University Hospital and Faculty of Medicine of Masaryk University, Brno, Czech Republic; 4 Genomics Laboratory, MRC Clinical Sciences Centre, Imperial College, London, United Kingdom; 5 National Heart Centre Singapore, Singapore, Singapore; Medical University Hamburg, University Heart Center, Germany

## Abstract

**Background:**

Molecular genetic testing is recommended for diagnosis of inherited cardiac disease, to guide prognosis and treatment, but access is often limited by cost and availability. Recently introduced high-throughput bench-top DNA sequencing platforms have the potential to overcome these limitations.

**Methodology/Principal Findings:**

We evaluated two next-generation sequencing (NGS) platforms for molecular diagnostics. The protein-coding regions of six genes associated with inherited arrhythmia syndromes were amplified from 15 human samples using parallelised multiplex PCR (Access Array, Fluidigm), and sequenced on the MiSeq (Illumina) and Ion Torrent PGM (Life Technologies). Overall, 97.9% of the target was sequenced adequately for variant calling on the MiSeq, and 96.8% on the Ion Torrent PGM. Regions missed tended to be of high GC-content, and most were problematic for both platforms. Variant calling was assessed using 107 variants detected using Sanger sequencing: within adequately sequenced regions, variant calling on both platforms was highly accurate (Sensitivity: MiSeq 100%, PGM 99.1%. Positive predictive value: MiSeq 95.9%, PGM 95.5%). At the time of the study the Ion Torrent PGM had a lower capital cost and individual runs were cheaper and faster. The MiSeq had a higher capacity (requiring fewer runs), with reduced hands-on time and simpler laboratory workflows. Both provide significant cost and time savings over conventional methods, even allowing for adjunct Sanger sequencing to validate findings and sequence exons missed by NGS.

**Conclusions/Significance:**

MiSeq and Ion Torrent PGM both provide accurate variant detection as part of a PCR-based molecular diagnostic workflow, and provide alternative platforms for molecular diagnosis of inherited cardiac conditions. Though there were performance differences at this throughput, platforms differed primarily in terms of cost, scalability, protocol stability and ease of use. Compared with current molecular genetic diagnostic tests for inherited cardiac arrhythmias, these NGS approaches are faster, less expensive, and yet more comprehensive.

## Introduction

Molecular diagnostics are recommended in the management of inherited diseases, for diagnosis and stratified therapy [1], [2], [3], [4], but in practice are under-used due to issues of cost, time and availability of services. Next Generation Sequencing (NGS) DNA analysis technologies have the potential to overcome these issues [5]. Inherited cardiac conditions (ICC), such as inherited arrhythmia syndromes and cardiomyopathies, have been identified as a suitable area to pilot the development of NGS assays for clinical use [6], [7]. This is due to the relatively high burden of disease in the population and limitations of current diagnostic approaches in genetically heterogeneous conditions such as these.

A number of bench-top NGS platforms have recently been introduced capable of Gigabase-scale DNA sequencing with relatively short run times (<27 hrs), including the MiSeq (Illumina) and the Ion Torrent Personal Genome Machine (PGM; Life Technologies). Initial studies have used these to characterise genetic targets of clinical significance including; bacterial genomes [8], [9], [10], [11], [12], [13], [14], [15], [16], the human breast cancer *BRCA* gene [11], [17], the cystic fibrosis *CFTR* gene [18], HLA type [19] and somatic variation in cancer [20]. The high analytical throughput and relative speed make NGS assays very attractive for early clinical implementation, requiring an in-depth understanding of the strengths and limitations of each platform in a clinical diagnostic setting.

A recent study by Loman *et al* [10] compared bench-top NGS platforms for sequencing *E.coli* genomes, which have a GC-content of 50%, during an outbreak investigation. They identified a higher rate of homopolymer-associated indel errors in raw reads when comparing the Ion Torrent PGM to the MiSeq (1.5 and <0.001 errors per 100 bases, respectively). The MiSeq also detected fewer single-base substitutions than the Ion Torrent PGM. A further recent study by Quail *et al* [8] performed similar analyses using a number of different bacterial reference genomes representing a range of GC-contents, including the *B.pertussis* genome which has a GC-content of ∼68% with some sub-genomic regions >90%. They observed a higher substitution error rate when using Ion Torrent PGM than the MiSeq platform (1.78 and 0.4 errors per 100 bases, respectively). Again, they reported fewer homopolymer-associated errors in MiSeq data than the Ion Torrent PGM. More variants were called using the Ion Torrent PGM versus MiSeq; however, this resulted in a slight increase in the number of false positive calls using the Ion Torrent PGM platform. Both NGS platforms generated adequate coverage across templates even in sub-genomic regions of very high GC-content. Significant efforts to improve sequencing performance and bioinformatics processing have been undertaken both by the bench-top sequencer manufacturers and the NGS community.

In this study, we used microfluidic multiplex PCR and NGS to sequence six genes that cause inherited arrhythmia syndromes in a panel of well characterised patient-derived genomic DNAs. We compared the performance of two bench-top MiSeq and Ion Torrent PGM DNA sequencing platforms, aiming to develop a comprehensive pipeline applicable to clinical diagnostics.

## Materials and Methods

### Human Specimens

The Hammersmith and Queen Charlotte’s & Chelsea Research Ethics Committee approved the study. DNA was obtained from subjects who had given written informed consent and was provided in accordance with Human Tissue Act, UK guidelines. Fifteen anonymised DNA samples were selected for technical assay evaluation. Eleven (group I) had undergone mutation scanning of five Long QT syndrome (LQT) associated genes (See Table 1) using denaturing high performance liquid chromatography (dHPLC) [21] coupled with Sanger DNA sequence analysis to confirm putative variants. Four (group II) underwent exon PCR amplification and direct Sanger DNA sequence analysis of the full coding sequence of the same five genes.

**Table 1:** Characteristics of six genes included in the assay.

Gene symbol	Gene product	Genomic locus	^1^Reference sequence	Number of exons	Coding cDNA length (bp)	Number of amplicons	Disease association
*KCNQ1*	I_Ks_ channel, alpha subunit	11p15.5	LRG_285_t1	16	2015	30	LQT1
*KCNH2*	I_Kr_ channel, alpha subunit	7q36.1	LRG_288_t1	15	3465	54	LQT2
*SCN5A*	Na_v_1.5 channel, alpha subunit	3p21	LRG_289_t1	27	6024	78	LQT3
*KCNE1*	I_Ks_ channel, beta subunit	21q22.12	LRG_290_t1	1	389	4	LQT5
*KCNE2*	I_Kr_ channel, beta subunit	21q22.12	LRG_291_t1	1	371	5	LQT6
*RYR2*	Sarcoplasmic reticulum calcium-induced calcium release	1q43	LRG_402_t1	104	14,785	215	CPVT
Total				164	27,049	386	

LRG: locus reference genomic (http://www.lrg-sequence.org), LQT: Long QT syndrome, CPVT: catecholaminergic polymorphic ventricular tachycardia.

### Target Enrichment by PCR Capture

Initial Access Array primer design was undertaken by Fluidigm Corp. (South San Francisco, CA) using the Primer3 oligonucleotide design tool [22]. Prior to this study the assay was further optimized in-house, with additional primers designed to target regions that were not well captured in pilot studies using the Ion Torrent PGM [23]. In the final assay 386 amplicons targeted the protein-coding sequence of six inherited arrhythmia genes (Table 1), with an overhang at exon boundaries to capture splice site variants. Figure S1 in the Supporting Information illustrates the GC-content and length distribution of the 386 Access Array amplicons.

Genomic DNA templates were amplified using the 48.480 Access Array IFC, according to the manufacturer’s instructions (Fluidigm). In brief, each sample DNA was combined with primer pairs in a microfluidic chip, with a maximum capacity of 48 samples×48 10-plex reactions. The chip was loaded with PCR reagents and transferred to a thermocycler. Common flanking sequences (CS) on each primer pair permit attachment of platform-specific barcode indexes and sequencing adaptors in a subsequent fusion PCR. Pooled amplicons from each DNA template were harvested and used as input for platform-specific library preparation.

### Platform-specific Barcode/Adapter Attachment

For MiSeq, we followed standard Fluidigm protocols. Amplicons were diluted 1∶100 and subjected to a single fusion PCR reaction using the bidirectional 386 barcode kit, with the FastStart High Fidelity Enzyme kit (Roche), as per manufacturer’s instructions. A unidirectional library was prepared for paired-end sequencing: for each reaction, 1 µl of the diluted harvested PCR pool was mixed with forward “A” barcodes (indexes 1 to 15, final concentration 400 nM) and 15µl of PCR pre-mix. Cycling conditions were as follows: initial incubation at 95°C for 10 min; 15 cycles of 95°C for 15 sec, 60°C for 30 sec and 72°C for 1 min; final incubation at 72°C for 3 min; hold at 4°C.

For Ion Torrent PGM, commercial barcoding protocols were not available at the time of the study, so we employed an equivalent fusion PCR approach using custom oligonucleotides, yielding a 10 base pair (bp) barcode and Ion Torrent PGM adaptor (Table S1 in the Supporting Information). The amplicon harvest volume was adjusted to 20µl using PCR certified water, and two barcode-fusion PCR reactions were prepared using opposing CS-tagged primer pairs (e.g. pairing A_BC6_CS1 with CS2_P1, and A_BC6_CS2 with CS1_P1). This strategy permitted sequencing of each amplicon in both orientations, *in lieu* of paired-end sequencing. For each reaction, 10 µl of the Fluidigm harvest was added to 86 µl of a Herculase II Fusion PCR mix, as per manufacturer’s instructions (Agilent Technologies Inc, Santa Clara, CA) along with 20 pmol each primer. Cycling conditions were as follows: initial incubation at 98°C for 30 sec; two cycles of 98°C for 30 sec, 54°C for 30 sec and 72°C for 30 sec; final incubation at 72°C for 2 min; hold at 4°C.

### MiSeq Sequencing

MiSeq sequencing was performed at the MRC Clinical Sciences Centre Genomics Laboratory, Imperial College London, using MiSeq Reagent kit v1, MCS v1.1.1 and RTA v1.13.56 for performing image analysis, base calling and quality control (QC).

### Ion Torrent PGM Sequencing

Ion torrent PGM sequencing was completed at Royal Brompton Hospital using Ion One Touch 200 reagents kits (Release: 20 February 2012, Rev. C), Ion PGM 200 Sequencing Kit (Release: 21 February 2012, Rev. B) and 316 scale chips. Sequence analysis was completed with Ion Torrent Suite 2.2 (ITS2.2; Life Technologies) packages. Sequence analysis and variant calling were subsequently repeated using ITS3.2, but the results were unchanged, and data from ITS2.2 is presented here.

### Bioinformatic Primer Trimming and Read Mapping

Default parameters were used for all data processing and analysis stages unless otherwise specified. FastQC version 0.9.5 (http://www.bioinformatics.babraham.ac.uk/projects/fastqc/) was used to assess sequence quality metrics for each sample, including per-base and per-sequence quality scores, GC-content, and read length distribution. Raw sequences generated by MiSeq and Ion Torrent PGM included primer sequences at both 5′ and 3′ ends. For MiSeq data, primers were trimmed using an in-house Perl script, before quality control (average base quality in a 30 bp sliding window >20; 3′ read trimming of bases with a quality score <6; removal of reads <20 bp in length) and alignment with BWA (version 0.6.1-r112-master) [24]. Figure S2 and S3 in the Supporting Information demonstrates the base quality and length distribution before and after primer trimming for one sample from MiSeq. Ion Torrent PGM reads were aligned using ITS2.2, incorporating tmap (version 0.3.7). The variantCaller plugin module trimmed primers using an aligned bam file intersected with an amplicon-only bed file. Human genome reference sequence (hg19) was used for both platforms.

Coverage of the target was assessed using BedTools [25]. The number of bases covered at sufficient depth and quality for variant calling was assessed using the Genome Analysis Toolkit (GATK; version 1.5) [26] Callable Loci Walker. Evenness was calculated according to the method described by Mokry *et al* [27] and implemented with the R statistical package (http://www.r-project.org). This yielded a score in the range 0–1, with 1 indicating uniform coverage. Target enrichment factor (EF) was calculated as, 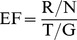, here R represents the reads on target; N represents total mapped reads; T represents target size and G represents genome size [28].

### Variant Detection

MiSeq reads were processed using Picard tools (version 1.65, http://picard.sourceforge.net) and Samtools (version 0.1.18) [29], and variants were called with GATK. A standard GATK pipeline was applied including realignment around known indels (dbSNP135) and recalibration. All reads were used for variant calling, without downsampling or removal of PCR duplicates. Variants with QD <5 or MQ <30 or DP<30 were filtered out. For the Ion Torrent PGM, variants were called using the ITS2.2 variantCaller plugin with the Ampliseq and germline workflow. Primers were trimmed and variants called with a variant frequency threshold at 25%. The Integrative Genomics Viewer (IGV) [30] was used for visualization.

### Reference Comparator by Sanger DNA Sequencing

Direct dideoxy Sanger DNA sequencing was used to sequence all protein coding regions of five LQT genes in samples from group II. Amplicons were prepared using Platinum *Taq* PCR (Life Technologies) and GC-Rich PCR system (Roche), and sequenced using the ABI 3730XL DNA analyzer (Life Technologies). Though sequenced by NGS, *RYR2* was not included in comparisons as its large size made validation prohibitive. DNA sequence analysis was performed using Sequencher 4.10.1 (Genecodes Inc, Ann Arbor, MI). Any discordant variant calls between NGS and dHPLC in group I were also confirmed by Sanger sequencing. The total number of bases sequenced by the direct Sanger DNA sequencing method was 61,380 bp.

The sensitivity and positive predictive value (PPV) of variant detection were calculated by comparing the gold-standard Sanger data to the NGS data for each platform. 95% confidence intervals (CI) were calculated using Jeffreys interval, implemented in the *binom* package in R.

## Results

### Sequencing Data Output and Quality

Total sequencing output and mean read lengths from the two approaches were comparable (Table 2). A single MiSeq run produced 8.13 million reads (1230 Mb of sequence) as compared to three 316 chip-scale Ion Torrent PGM runs that generated 6.56 million reads (1001 Mb of sequence). Raw reads generated by MiSeq have 151 bp fixed length (paired-end read), whilst reads generated by Ion Torrent PGM had a variable length, using the 200 bp Ion Torrent PGM chemistry kits. The average length of reads in three Ion Torrent PGM runs was 150 bp (single-end read).

**Table 2:** Comparison of bench-top NGS platforms.

	MiSeq	Ion Torrent PGM*
NGS runs	1	3
Template preparation	1 hr	3×5.5 hr
Run time	27 hr	3×3 hr
Barcodes	15 (commercial)	3×5 (custom)
Theoretical sequencing output	1.5 Gb	3×1.27 Gb
Actual sequencing output	1.23 Gb	1.00 Gb
Number of sequencing reads	8.13 M	6.56 M
Read length output	151	150**
Paired-end reads	Yes	No
Instrument cost	$125k	$75k
Sequencing cost for assay	$959	3x$686
Per specimen sequencing cost	$64	$137

* 316 scale chip; ** average.

The two platforms produced a similar yield of filtered sequence bases. The MiSeq platform produced 95.8% high quality (Q20) bases and the Ion Torrent PGM 67.5%. As platforms use different algorithms to estimate base quality, apply different downstream quality filters and call variants differently [31], [32], these raw quality scores are most useful for comparing runs within platform, and are provided here for comparison to other datasets. We do not use these data to compare sequencing performance between platforms. For the MiSeq, raw reads contained primer sequences and lower quality bases at the 3′ ends. Primer trimming and quality control discarded 4.6% of reads, and excluded 27.6% of bases; only 18.4% of Q20 bases were excluded, and final trimmed reads comprised 95.8% Q20 bases. 90.7% of the trimmed reads mapped to the reference genome, and 96.7% of these mapped reads were on-target. The average depth of coverage on-target was 1529-fold (Table 3), with evenness 0.68 and EF 110111.

**Table 3:** Sequencing and target capture performance metrics.

					Alignment						
NGS Platform	Reads	Bases (Mb)	Mean read length	Q20 Bases	Mapped Reads	Reads On Design	Reads On Target	Depth On Target	Evenness	EF	Callable
MiSeq	7757916	889	115	95.8%	90.7%	99.3%	96.7%	1529	68.1%	110111	97.9%
PGM	6133098	969	106	67.5%	100%	96.2%	91.2%	1231	78.8%	104915	96.8%

Mean read length after trimming primer sequences and low quality bases. ReadsOnDesign/ReadsOnTarget = percentage of reads mapping to amplicon design or protein-coding target region. EF = enrichment factor.

For the Ion Torrent PGM, reads still contained primer sequences after de-multiplexing, thus primer trimming was performed following the alignment procedure. We observed 93.5% raw reads were mapped to the reference genome; 91.2% of the mapped reads were on-target. The average depth of coverage on-target was 1231-fold across all samples (Table 3) with evenness 0.79 and EF 104915. Both evenness and enrichment factor differed significantly between platforms (p-value <2.2×10^−16^ for evenness and p-value<3.3×10^−6^ for EF; paired t-test).

### Target Enrichment Performance

**Figure 1:**
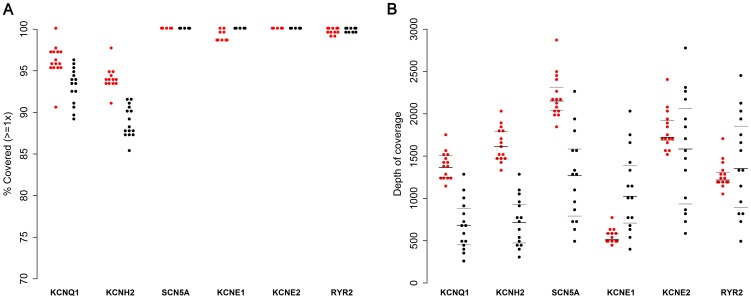
Coverage of target genes. a. The percentage of each gene that is captured and sequenced (at least one read) is shown for each platform (MiSeq in red, PGM in black), for 15 samples; Three genes were consistently fully sequenced. Coverage of KCNQ1 and KCNH2 was more variable: KCNQ1 and KCNE1 were fully covered in the best performing samples, while the best performance on KCNH2 covered >97% of the gene. b. Mean sequencing depth across each gene, for 15 samples. Quartiles are shown. There is significant intra- and inter- sample variability.

Figure 1 summarises the coverage of our genes of interest for each platform. Overall, 98.8% of the target region was covered by at least one read for the MiSeq and 98.0% for the Ion Torrent PGM (Table 4 and Figure S4 in Supporting Information). For three genes (*SCN5A1, KCNE2* and *RYR2*) coverage consistently approached 100% on both platforms. *KCNQ1* and *KCNH2* were less consistently well covered, averaging 96.2% and 94.1% for MiSeq, and 93.1% and 88.9% for Ion Torrent PGM, respectively. While coverage at 1× was almost complete for *KCNE1*, part of the gene achieved consistently low sequencing depth on the MiSeq only (see Discussion).

**Table 4:** Sequencing coverage of each gene.

	MiSeq (%)						PGM (%)					
	1x	10x	20x	30x	50x	100x	1x	10x	20x	30x	50x	100x
***KCNE1***	98.83	88.77	75.2	69.44	67.64	65.5	100	100	100	100	100	99.81
***KCNE2***	100	96.42	88.93	85.98	85.98	85.98	100	100	100	100	100	100
***KCNH2***	94.07	87.66	85.67	84.3	83.22	81.79	88.86	77.91	75.63	74.51	72.95	70.59
***KCNQ1***	96.22	92.86	89	86.42	79.56	74.03	93.14	86.45	82.68	81.42	80.92	77.39
***SCN5A***	100	100	99.98	99.69	98.72	95.96	100	100	99.96	99.9	99.75	99.13
***RYR2***	99.69	97.2	95.16	93.7	90.69	85.78	99.87	99.67	99.65	99.61	99.38	98.86
**Overall**	98.77	96.14	94.19	92.83	90.3	86.37	97.99	95.98	95.39	95.12	94.72	93.73

The coverage of the protein-coding sequence of each gene of interest is tabulated, as a percentage, for a range of sequencing depths (≥1x, 10x, 20x, 30x, 50x and 100x reads).

The mean coverage of the protein-coding region of every gene was consistently >200 reads on both sequencing platforms (Fig. 1b). The depth of coverage was more consistent between samples on the MiSeq than on the Ion Torrent PGM (Figure 1b). By contrast, within-sample coverage was more consistent on the Ion Torrent PGM (evenness 0.78 vs. 0.68, p<2.2×10^−16^; Table 3). While the MiSeq provided deeper coverage overall, *KCNE1* was an outlier on this platform (Figure 1b), suggesting a platform-specific sequencing difficulty (See Discussion).

**Figure 2:**
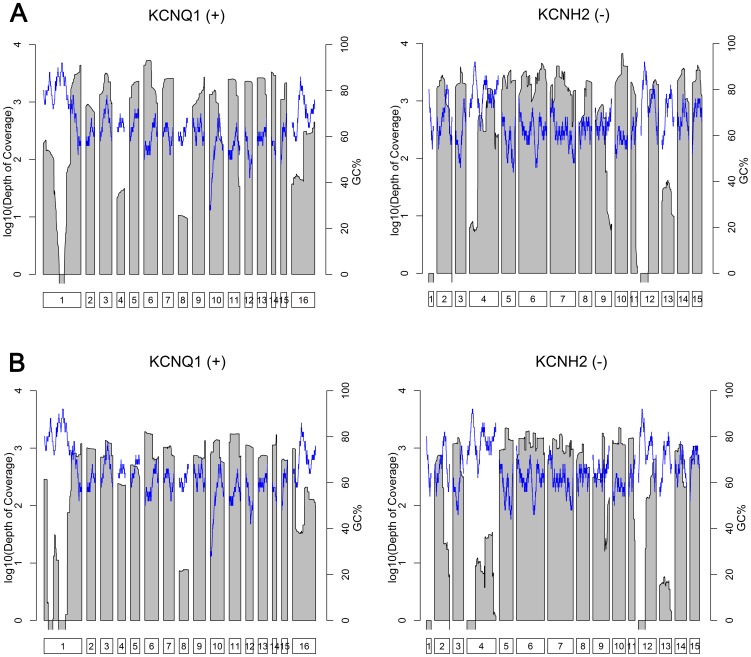
Coverage of KCNQ1 and KCNH2 for the two platforms. Mean depth of coverage for 15 samples is shown for two genes on a log scale. Regions of no coverage therefore have negative values. The blue lines indicate local GC content (calculated with a 50 bp sliding window). Regions consistently missed have high GC content, with similar patterns for both platforms. KCNQ1 exons 1 & 8 and KCNH2 exons 1, 4 & 12 are difficult to sequence. A cartoon of the exon structure is shown beneath each panel. Plus (+) and minus (-) denote gene strand. Plots for all genes are shown in Supporting Information Figure S5. a.) MiSeq b.) Ion Torrent PGM.

The influence of GC-content on performance was assessed. GC-content was calculated using a 50 bp sliding window and plotted alongside sequencing depth across the target for each NGS platform (*KCNQ1* and *KCNH2* are shown in Figure 2, remaining genes in Supporting Information Figure S5). We found that both platforms performed less well in regions of very high GC-content (*KCNQ1* exons 1 & 8, *KCNH2* exons 1, 4, 12, and the 3′ portion of exon 2). The relationship between GC-content and performance was most reproducible for the Ion Torrent PGM. While the MiSeq displayed more variability in sequencing depth (See Table 3), the relationship with GC was weaker suggesting that other factors may be limiting (Figure S6 in the Supporting Information).

### Variant Detection

Variant detection was assessed using a panel of variants previously identified by dHPLC mutation scanning (group I) or Sanger sequencing (group II) (Table 5). The majority of known variants were detected on both platforms, with a small number of variants missed by each. NGS platforms also detected a number of variants not previously identified, mainly in samples where dHPLC rather than Sanger sequencing was used for initial variant detection. In these samples, validation by Sanger sequencing confirmed 28/32 (87.5%) unexpected MiSeq variants, and 33/36 (91.7%) Ion Torrent PGM variants (Table 6), which were therefore dHPLC false negatives. The MiSeq produced four genuine false positive (FP) SNP calls, and the Ion Torrent PGM five FPs (four SNPs and one indel), equivalent to positive predictive values (PPV) of 95.9% (MiSeq; 95% CI 90.5–98.6%) and 95.4% (PGM; 95% CI 90.3–98.2%).

**Table 5:** Detection of coding variants for each NGS platform.

	Variants detected by dHPLC/Sanger	Variants detected by MiSeq			Variants missed by MiSeq		Variants detected by PGM		Variants missed by PGM
		Total	Concordant with dHPLC/Sanger	NGS only		Total	Concordant with dHPLC/Sanger	NGS only	
Group I(dHPLC)	38	64	32	32	6	74	38	36	0
Group II(Sanger)	36	33	33	0	3	36	34	2	2
Total	74	97	65	32	9	110	72	38	2

**Table 6:** Accuracy of variant calling for NGS platforms.

	Total coding variants	Variant sites interrogated	Variants detected	False positives	Variants missed	Sensitivity	Positive predictive value
**MiSeq**							
Group I	71	60	60	4	0	100%	93.8%
Group II	36	33	33	0	0	100%	100%
**Total**	**107**	**93**	**93**	**4**	**0**	**100%** **(98.0–100)**	**95.9%** **(90.5–98.6)**
**PGM**							
Group I	71	71	71	3	0	100%	96.0%
Group II	36	35	34	2	1	97.1%	94.4%
**Total**	**107**	**106**	**105**	**5**	**1**	**99.1%** **(95.7–99.9)**	**95.5%** **(90.3–98.2)**

Group I: dHPLC with Sanger confirmation; Group II: direct Sanger sequencing 95% confidence intervals are given for sensitivity and positive predictive value.

Variants not detected by NGS were primarily located in regions without any sequencing coverage. On the MiSeq platform, 14 known variants were not detected (see Table S2 in the Supporting Information). These included a single common polymorphism in *KCNE1* that was present in 12 samples (chr21∶35821821, Supporting Information Figure S4), and a separate SNP in the same gene (chr21∶35821795). This single exon gene was well covered by the Ion Torrent PGM, but consistently inadequately sequenced by the MiSeq across samples, suggesting a platform-specific, sequence context dependent limitation, rather than a failure of the upstream PCR capture. The final false negative on the MiSeq was also missed by the Ion Torrent PGM (chr7∶150645534, *KCNH2*) in same sample, with no sequencing reads on either platform at this region of high GC content (>70%), suggesting that the upstream PCR did not capture this region. The final variant missed by the Ion Torrent PGM was found in a well-captured region of *KCNQ1* (del at chr11∶2594088). Individual PGM reads contain a high rate of indels in hompolymer stretches, and the Bayesian calling algorithm has been optimised to eliminate these when calling variants in the consensus sequence. This true deletion (ACCACCCT -> ACCACCT) resembles such an error so, while putative variant alleles were detected by ITS, the variant was rejected as a probable error. This outcome is insensitive to user-defined filter settings.

Of variants located in sequenced regions, 93/93 (100%; 95% CI 98.0–100%) were detected by MiSeq, and 105/106 (99.1%; 95% CI 95.7–99.9%) by the Ion Torrent PGM (Table 6). In a diagnostic setting, the handful of regions of predictable and consistent low coverage (which harboured the missed variants) would be targeted with adjunct Sanger sequencing.

The study was not powered to formally compare indel calling, but 4/4 known indels were detected by MiSeq, and 3/4 by Ion Torrent PGM with one FP.

### Resources and Costs

The MiSeq data was obtained with a single sequencing run, at a cost of $959 (£609). It required three Ion Torrent PGM runs to obtain equivalent sequencing output ($686 each) (£439), and one run was repeated due to low bead deposition on the sequencing slide. The total sequencing time (from pooled, barcoded sequencing library to raw sequence data output) was 28 hrs for MiSeq, including one hour of hands-on time. The equivalent time for each Ion Torrent PGM run was shorter (9 hrs), but with 4 hrs hands-on time, as emulsion PCR, enrichment and sequencing occur on separate machines with human intervention at each stage, whereas chip loading and cluster generation are automated on the MiSeq (Table 2).

In summary, PCR-based target enrichment approach followed by MiSeq and Ion Torrent PGM sequencing interrogated 97.9% and 96.8% of the target sufficiently for variant detection with equivalent NGS sequencing output. Variant calling in the regions covered had a PPV of 95.9% (MiSeq, 95% CI: 90.5–98.6%) and 95.5% (PGM, 95% CI: 90.3–98.2%) with sensitivities of 100% (MiSeq, 95% CI: 98.0–100%) and 99.1% (PGM, 95% CI: 95.7–99.9%) (Table 6). In a diagnostic setting, the handful of regions missed are most likely to require adjunct Sanger sequencing to achieve up to 100% sensitivity for the assay as a whole.

## Discussion

### Assay Coverage

Both platforms achieved very good coverage of the target region. It is unlikely that such an assay will achieve 100% coverage, largely because GC-rich target is difficult to amplify using PCR, both at the target enrichment stage and also during downstream NGS library preparation. We anticipate that for diagnostic use a small number of regions will continue to require conventional sequencing approaches, though such a hybrid approach still provides for a cost and time saving compared with conventional sequencing. For the six genes studied here, there are 1256 reported disease-causing variants in this protein-coding target region [33]; 1217 (96.9%) fall within the regions covered by the MiSeq, and 1200 (95.5%) by the Ion Torrent PGM.

The Access Array design was iteratively optimized prior to this study (see Methods). The performance of the manufacturer’s original amplicon design was assessed, and additional primer pairs added to the assay to improve the capture of regions that were under-represented. This pilot work used the Ion Torrent PGM, as the MiSeq was not available in the UK at that time. This may marginally favour the Ion Torrent PGM: the MiSeq platform performed poorly on amplicons derived from *KCNE1* (chr21∶35821729–35821867, Supporting Information Figures S4 and S5), though these were captured by the Access Array. It may be possible to produce MiSeq-compatible amplicons with further iteration tailored to this platform.

These clinically important genes include regions with a very high GC-content (∼80%), such as *KCNQ1* exons 1 & 8, *KCNH2* exons 1, 4, 12, and the 3′ portion of exon 2, which perform relatively poorly despite optimization efforts. We have previously found that the performance of some amplicons in the Access Array can be improved using a GC robust PCR mastermix at this stage, but these gains are unlikely to persist if non-GC robust enzymes are used in downstream emulsion PCR during NGS library preparation. However, as Quail *et al* were able to successfully sequence sub-genomic regions with GC-contents >90%, upstream PCR capture, rather than NGS, is likely to be limiting here, and this avenue may still yield further improvements.

An alternative upstream target capture technology might also yield better coverage and hence sensitivity. In this study the capture methodology was fixed to allow unbiased comparison of downstream sequencing, but we have previously compared PCR and hybridisation based approaches for these same gene [34], and found that overall coverage was very similar for both approaches. Other studies have reported reproducible patterns of non-uniform capture across a range of platforms, particular in repetitive sequences and at extremes of GC content [34], [35]. In our opinion the choice of upstream target capture is most likely to be driven by cost and capacity requirements: the microfluidic PCR approach employed here is simple, fast and cheap, but has a much smaller capacity than hybridisation approaches, for example.

At the throughput employed in this study, both platforms had significant redundancy of sequencing depth, making them relatively robust to differences in sequencing depth within and between samples. If more samples were processed in a single run to increase throughput, the differences in coverage variability within- and between- samples may become limiting and influence platform choice. Inter-sample variability was most marked when using Ion Torrent PGM, as compared to the MiSeq. Variability between samples (See Figure 1b) is most likely due to stochastic error during pipetting and quantification leading to differences in DNA input at the sequencing stage. In our study there was no evidence of systematic barcode bias where this could be assessed on the PGM. Within sample variability is largely reproducible and sequence-dependent, and is a well-recognized feature of all target enrichment methodologies [34], [36], though sequence-dependent bias is present even in whole genome sequencing, without target enrichment.

We acknowledge that we have only studied a small number of genes here, as the assay was matched to the capacity of a PCR-based approach, and intended to reflect a typical clinical assay. Though a range of gene sizes and GC contents were represented, this may limit the generalizability of findings.

### Variant Calling

Variant calling was reassuringly accurate. Sensitivity in the regions covered by the assay was excellent with just one variant missed on one platform. Of the four FP SNPs from MiSeq and four FP SNPs from Ion Torrent PGM, one common error in *KCNH2* exon 5 (chr7∶150654468G>A) was called on both platforms. This site was deeply sequenced with good allele balance (sequencing depth 2730-fold with 57% alternate reads on MiSeq; sequencing depth 2403-fold with 55% alternate on PGM), good mapping quality and variant detection scores from both platforms. It was the only variant to be discordant between both NGS platforms and the Sanger method, raising the possibility that it is a sequence error introduced by upstream PCR. Five out of six remaining FP SNPs (three MiSeq, two Ion Torrent PGM) were G>A transitions clustered in *KCNH2* exons 12 and 13, and the final Ion Torrent PGM FP was a G>A transition in *SCN5A*. Ion Torrent PGM FPs occurred in regions with good sequencing depth, but significant strand imbalance and noisy sequencing (high base quality in individual reads, but poor consensus between reads). MiSeq FPs were found in areas of relatively low coverage (<100x), with false alternate allele bases found close to the ends of the reads, again with strand imbalance.

Importantly, our pipeline included a custom Perl script to trim poor quality bases at the 3′ end of MiSeq reads. This significantly improved the mapping qualities and reduced the number of false negatives on this platform in our hands (i.e. 9 common variants were rescued which would have otherwise been missed even with depth >400). Analysis of raw reads on both platforms showed a similar substitution mismatch rate (0.5 per 100 bases), with a higher indel rate in homopolymer stretches on the Ion Torrent PGM (1.3 vs 0.02 per 100 bases). Nonetheless, final variant calling accuracy did not differ significantly (odds ratio = 0.90; 95% confidence interval: 0.24–3.46; p-value = 1; Fisher’s exact test). This study was not powered to robustly assess differences in indel detection.

The number of PCR amplification cycles used in the two methodological approaches differed slightly. The MiSeq method used 76 PCR amplification cycles, including 26 cycles during flowcell cluster generation, whereas the Ion Torrent PGM used 82 cycles of PCR amplification, including 45 cycles during emulsion PCR. Increasing the number of PCR amplification cycles is known to increase the burden of *Taq*-related errors [37]. There may be room to reduce the number of cycles: for example the manufacturer’s protocol for Illumina library preparation uses a small aliquot of diluted template from the Access Array, removing this dilution may allow for fewer PCR cycles.

Current practice in laboratories that are starting to use NGS for clinical applications is to confirm medically actionable variants using Sanger sequencing. This study identified a small but significant number of false positives on both platforms, supporting this practice.

### Cost and Time

Given the strong technical performance of both platforms, issues of cost and time are likely to be important to laboratories. Sequencing capacity and costs change continuously as NGS platforms evolve, but at present the instrument cost of the MiSeq is higher than the Ion Torrent PGM. For a single run, the Ion Torrent PGM was cheaper and faster than the MiSeq, but with more hands-on time and a higher degree of technical complexity. With the throughput used in this study, the final cost per sample was lower for the MiSeq.

The relative youth of the Ion Torrent PGM (UK commercialisation date: mid-2011) means that it is developing rapidly, offering both advantages and challenges to early adopters. Challenges have included rapidly changing laboratory and bioinformatic protocols, reliability issues in our hands, and a modest per-run capacity at this stage. We readily acknowledge that performance on each platform is limited by user experience as well as platform capability, and therefore is likely to continue to improve. Positive developments include the semi-automation of emulsion PCR and bead enrichment, with reduced hands-on time, and the introduction of a larger scale 318-chip, with the potential to match the data output of the MiSeq in a single run. These changes may make the Ion Torrent PGM faster and cheaper overall, though still with more hands-on time than MiSeq. Though we have piloted the 318-scale chip with satisfactory sequencing and quality metrics (data not shown), at the time of data collection for this study we had not yet achieved balanced sequencing of multiple libraries in order to make use of the increased capacity and were continuing to use the 316. Subjectively, the MiSeq (UK commercialisation date: early 2012) has presented a shallower learning curve, with relatively stable protocols and software around the study period. When using the MiSeq platform to sequence low complexity libraries, sequence quality metrics and the number of reads passing bioinformatic filters are noticeably worse than those obtained during high-complexity genome sequencing. Illumina recommend adding 40–50% of a high complexity target (e.g. phi-X bacteriophage genomic DNA) to low complexity PCR-generated libraries at the sample loading stage. This may benefit smaller Access Array-generated libraries, or libraries with fewer samples in the multiplex. Whilst not used for this study, this practice would impact on the total useable yield of the MiSeq platform if widely adopted.

Current diagnostic testing for inherited cardiac arrhythmias in the United Kingdom is limited to a small number of laboratories, using exon PCR and direct Sanger sequencing or first-generation NGS DNA sequencing techniques. We are aware of one UK centre offering NGS analysis of the 5 LQT genes studied here (plus *KCNJ2*) on the Roche 454 GS-FLX sequencer with advertised turnaround time of 40 working days at a cost of $950 (£600) per specimen. The 454 currently produces fewer reads than the desktop sequencers studied here, and the high-throughput target-enrichment approach that we have employed does not require the longer read-lengths that are considered one of the principle advantages of this platform. We conservatively estimate that a diagnostic workflow using multiplex PCR and desktop NGS takes 20 working days to complete (including variant confirmation by Sanger sequencing), with likely cost of less than $630 (£400) per specimen if demand is sufficient to sequence at close to full capacity (full economic cost including DNA extraction, 15-plex testing with MiSeq NGS and Sanger variant confirmation studies). The assay described here also includes the large *RYR2* gene that is associated with another important inherited arrhythmia syndrome, catecholaminergic polymorphic VT (CPVT). *RYR2* is not currently fully sequenced in available clinical assays in the UK: testing is limited to “hotspot” exons (UK Genetic Testing Network, http://www.ukgtn.nhs.uk/, accessed 19^th^ February 2013). A combined assay for LQT & CPVT allows for higher assay throughput with reduced cost, and is sensible given the phenotypic similarity, the small but important number of *RYR2* mutations reported in “genotype-negative” LQT cases [38], and the value of comprehensive genetic testing in molecular autopsy.

In conclusion, we compared two NGS platforms for diagnostic sequencing. Whilst we do not recommend one platform over another, both are mature technologies for clinical application, with the potential to increase availability of molecular diagnostics in line with national and international recommendations. Performance is promising, though sequence-context and platform-specific biases will influence diagnostic strategies for some genes. Clinical labs should report the coverage of each gene interrogated by such an assay and use conventional methods to cover missed regions and to validate clinically actionable findings. The final choice of platforms is likely to be governed largely by cost and usability.

### Accession Numbers

Sequence data has been submitted to the European Nucleotide Archive, accession number ERP002466.

## Supporting Information

File S1 **ComparisonMiSeq_PGM_supplementary.docx includes six figures and two tables. Figure S1. Characteristics of target capture design: GC content and length of Access Array IFC amplicons.** a. Amplicon GC content approximates to a normal distribution 50.3±11.4 (%), <7% amplicons have extreme (>70% or <30%) GC-content. b. Amplicon length (range: 65 bp to 403 bp, median 190 bp and mean 185±29); 85% have a length <200 bp; 98% amplicons have sequence length <240 bp. We used optimised Fluidigm capture to prepare library for Illumina and Ion Torrent platforms (see methods). 386 amplicons, with a combined length of 71,915 bp, are tiled over 47,660 bp of target sequence, of which 27,049 bp is protein coding. **Figure S2. Base quality distributions.** Sequencing base qualities before (left) and after (right) trimming and QC from (a.) MiSeq. (b.) Ion Torrent PGM. The base quality distribution (boxplot at each bar) is plotting against position in the read; the solid-line curve indicates the average base quality. Reads from Ion Torrent PGM have better base quality at 3′end as compared to the raw reads generated by MiSeq. **Figure S3.Readlength distribution.** The read length from MiSeq (a) vary from 20 to 135 bp, with average 115 bp±26 and median 127 bp; Ion Torrent PGM produced up to 267 bp reads (b), with average 106 bp±57 and median 102 bp. **Figure S4. Coverage of target genes.** Here we show the percentage of each target gene that is covered at ≥ x sequencing depth, calculated as a mean across all samples.The lower panels show the same data, with a larger scale on the x-axis. On the PGM, two genes (KCNQ1 & KCHN2) show a sharp drop-off in coverage, suggesting that some regions are difficult to robustly sequence. On the MiSeq, KCNE1 and KCNE2 also showed significant drop-off. **Figure S5. Sequencing coverage of target genes.** Sequencing depth is plotted for each coding base of the six target genes, on a log_10_ scale. Depth is calculated as a mean across 15 samples. Regions covered by a single read are therefore plotted at the origin, and regions of zero coverage have a negative deflection on the y-axis. GC content (calculated with a 50 bp sliding window on the genomic DNA forward strand) is overlaid in blue. Plus (+) or minus (-) indicates the strand on which each gene is encoded. While some regions are clearly problematic for both platforms (e.g. KCNQ1 exon 2, KCNH2 exons 1 & 12), there are also regions where one platform performs better (e.g. KCNE1, KCNE2, KCNH2 exon 4). **Figure S6. The relationship between GC content and coverage.** Sequencing depth (log_10_ scale) for each exon is plotted against its GC content. The coefficient of variation is larger for MiSeq than for Ion Torrent PGM (0.931 vs. 0.407). Loess regression is shown in red. MiSeq performance appears more variable across the GC range, whereas Ion Torrent performance falls off at high GC values, perhaps because of the additional emulsion PCR. **Table S1. Barcode indexes and Ion Torrent specific adapters.** Primers used for Ion Torrent PGM barcoded library prep, with index sequences highlighted. Each amplicon is inserted into the complex in both orientations: A-adaptor_Barcode_CommonSequence1_Amplicon_CommonSequence2_P1-adaptor; A-adaptor_Barcode_CommonSequence2_Amplicon_CommonSequence1_P1-adaptor. **Table S2. Detected variant information.** LRG = Locus Reference Genomic; Chr = Chromosome; Ref = reference allele; Alt = Alternative allele; P = Variants revealed by PGM; M = variants revealed by Miseq; Highlighted indicates the SNP was missed by both platforms. Note: All variants appearing in this table were confirmed by Sanger DNA sequencing analysis.
(DOCX)
