# Supplementary material for: Towards Clinical Molecular Diagnosis of Inherited Cardiac Conditions: A Comparison of Bench-Top Genome DNA Sequencers
Source: PLoS One. 2013 Jul 4;8(7):e67744. doi: 10.1371/journal.pone.0067744 (PMC3701544; doi:10.1371/journal.pone.0067744)
Supplement: File S1 — ComparisonMiSeq_PGM_supplementary.docx includes six figures and two tables. Figure S1. Characteristics of target capture design: GC content and length of Access Array IFC amplicons. a. Amplicon GC content approximates to a normal distribution 50.3±11.4 (%), <7% amplicons have extreme (>70% or <30%) GC-content. b. Amplicon length (range: 65 bp to 403 bp, median 190 bp and mean 185±29); 85% have a length <200 bp; 98% amplicons have sequence length <240 bp. We used optimised Fluidigm capture to prepare library for Illumina and Ion Torrent platforms (see methods). 386 amplicons, with a combined length of 71,915 bp, are tiled over 47,660 bp of target sequence, of which 27,049 bp is protein coding. Figure S2. Base quality distributions. Sequencing base qualities before (left) and after (right) trimming and QC from (a.) MiSeq. (b.) Ion Torrent PGM. The base quality distribution (boxplot at each bar) is plotting against position in the read; the solid-line curve indicates the average base quality. Reads from Ion Torrent PGM have better base quality at 3′end as compared to the raw reads generated by MiSeq. Figure S3.Readlength distribution. The read length from MiSeq (a) vary from 20 to 135 bp, with average 115 bp±26 and median 127 bp; Ion Torrent PGM produced up to 267 bp reads (b), with average 106 bp±57 and median 102 bp. Figure S4. Coverage of target genes. Here we show the percentage of each target gene that is covered at ≥ x sequencing depth, calculated as a mean across all samples.The lower panels show the same data, with a larger scale on the x-axis. On the PGM, two genes (KCNQ1 & KCHN2) show a sharp drop-off in coverage, suggesting that some regions are difficult to robustly sequence. On the MiSeq, KCNE1 and KCNE2 also showed significant drop-off. Figure S5. Sequencing coverage of target genes. Sequencing depth is plotted for each coding base of the six target genes, on a log10 scale. Depth is calculated as a mean across 15 samples. Regions covered by a single read [file pone.0067744.s001.docx]

Molecular Diagnosis of Inherited Cardiac Conditions: A Comparison of Bench-Top Genome DNA Sequencers

**Figure S1. Characteristics of target capture design: GC content and length of Access Array IFC amplicons.**

**a.**


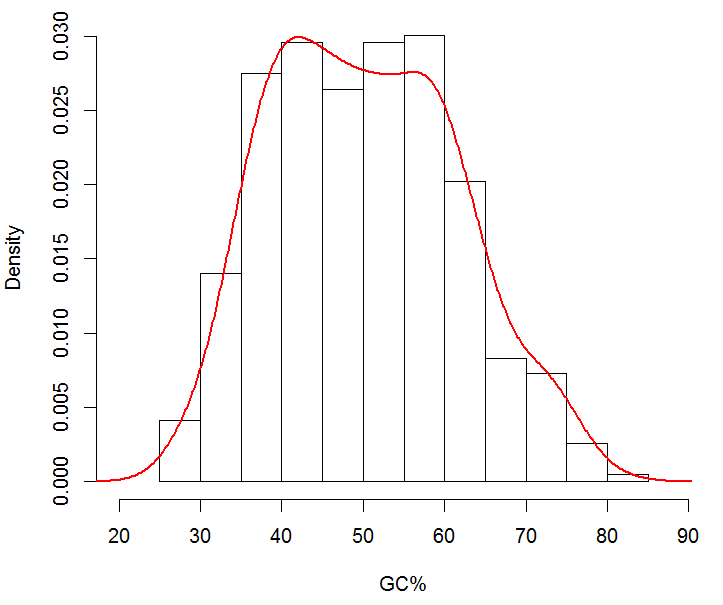


**b.**


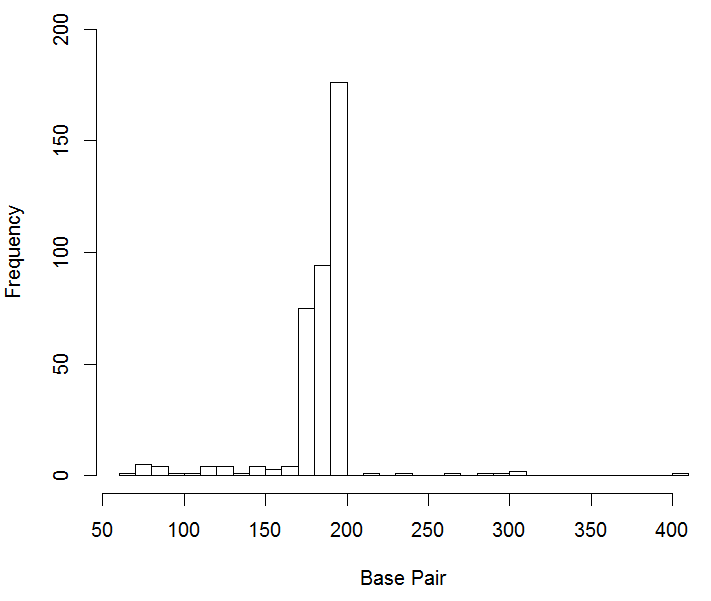


a. Amplicon GC content approximates to a normal distribution 50.3 ±11.4 (%), <7% amplicons have extreme (>70% or <30%) GC-content. b. Amplicon length (range: 65bp to 403bp, median 190bp and mean 185 ± 29); 85% have a length <200bp; 98% amplicons have sequence length <240bp. We used optimised Fluidigm capture to prepare library for Illumina and Ion Torrent platforms (see methods). 386 amplicons, with a combined length of 71,915bp, are tiled over 47,660bp of target sequence, of which 27,049bp is protein coding.

**Figure S2. Base quality distributions**

1. **Miseq**


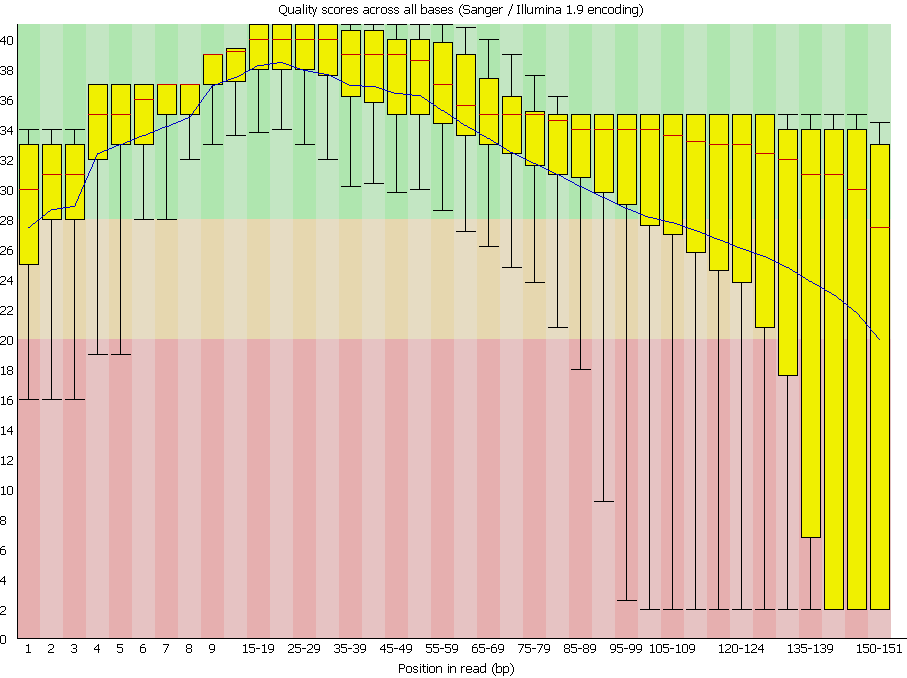

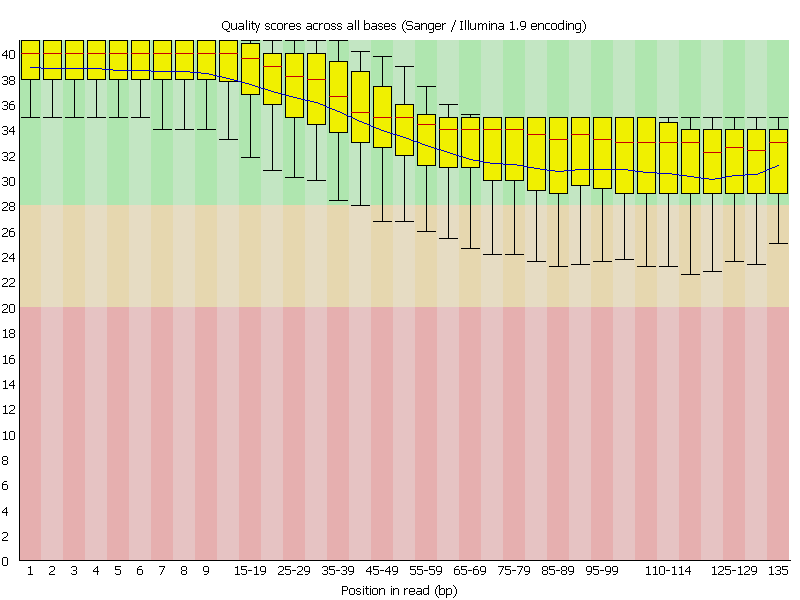


1. **Ion Torrent PGM**


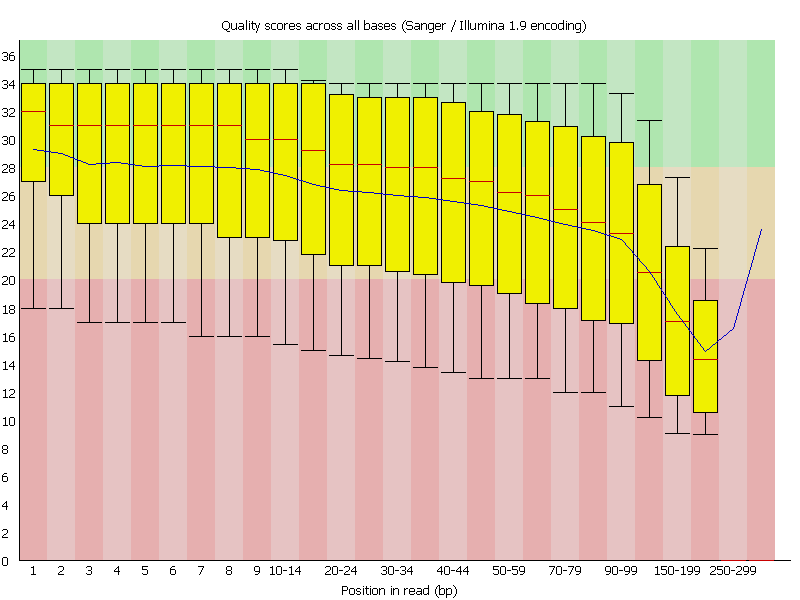

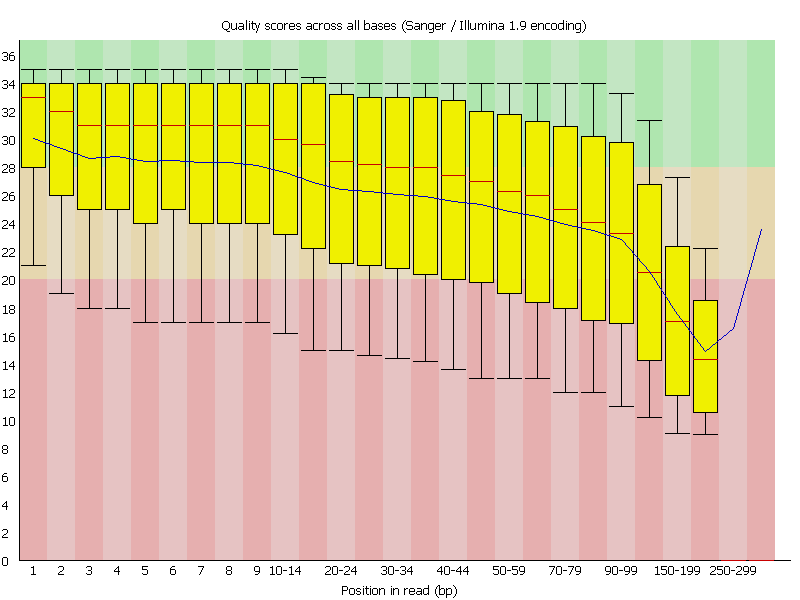


Sequencing base qualities before (left) and after (right) trimming and QC from (a.) MiSeq. (b.) Ion Torrent PGM. The base quality distribution (boxplot at each bar) is plotting against position in the read; the solid-line curve indicates the average base quality. Reads from Ion Torrent PGM have better base quality at 3’end as compared to the raw reads generated by MiSeq.

**Figure S3.Readlength distribution**

**a.**

**
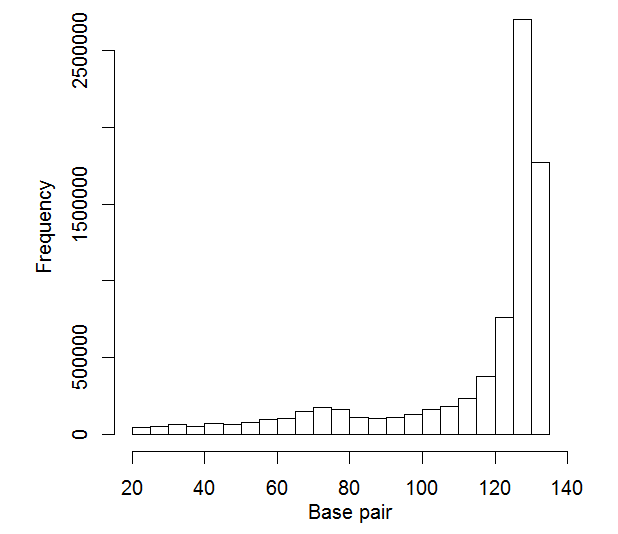
**

**b.**

**
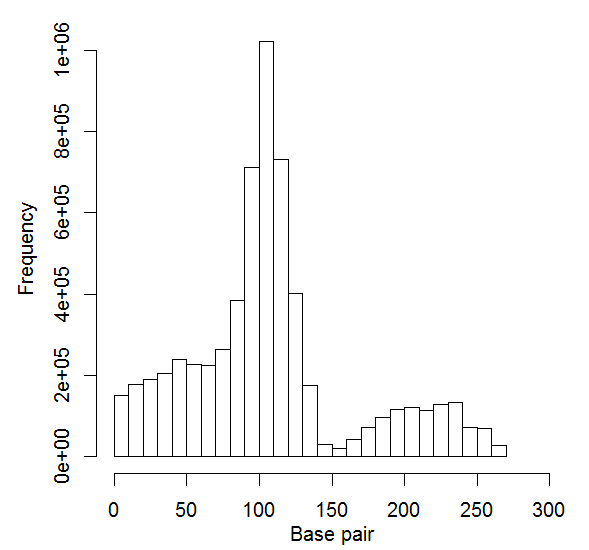
**

The read length from MiSeq (a) vary from 20 to 135bp, with average 115bp±26 and median 127bp; Ion Torrent PGM produced up to 267bp reads (b), with average 106bp±57and median 102bp.

**Figure S4. Coverage of target genes**

**
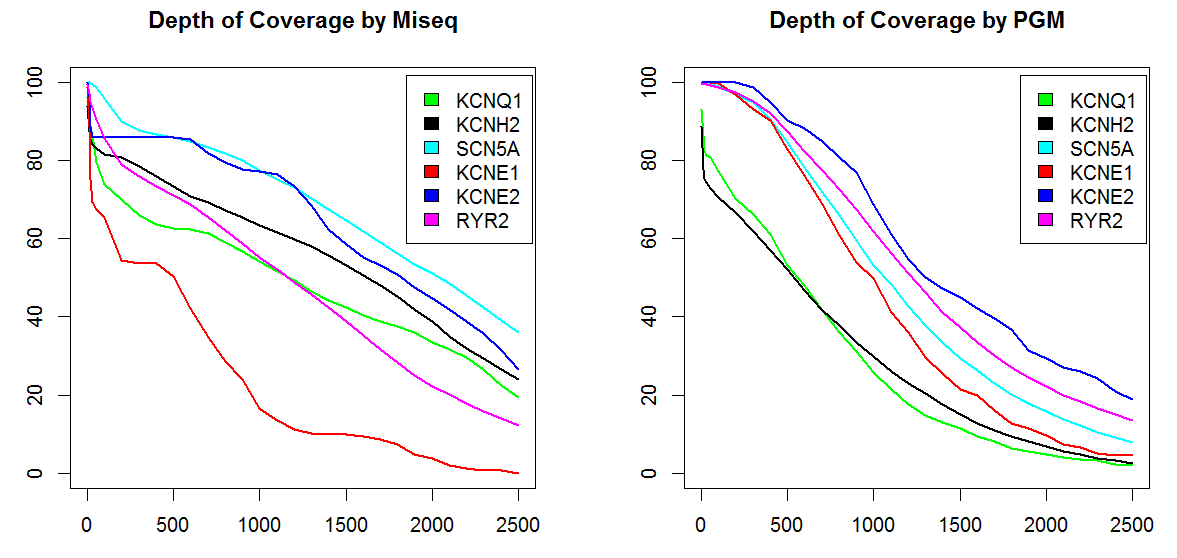
**

**
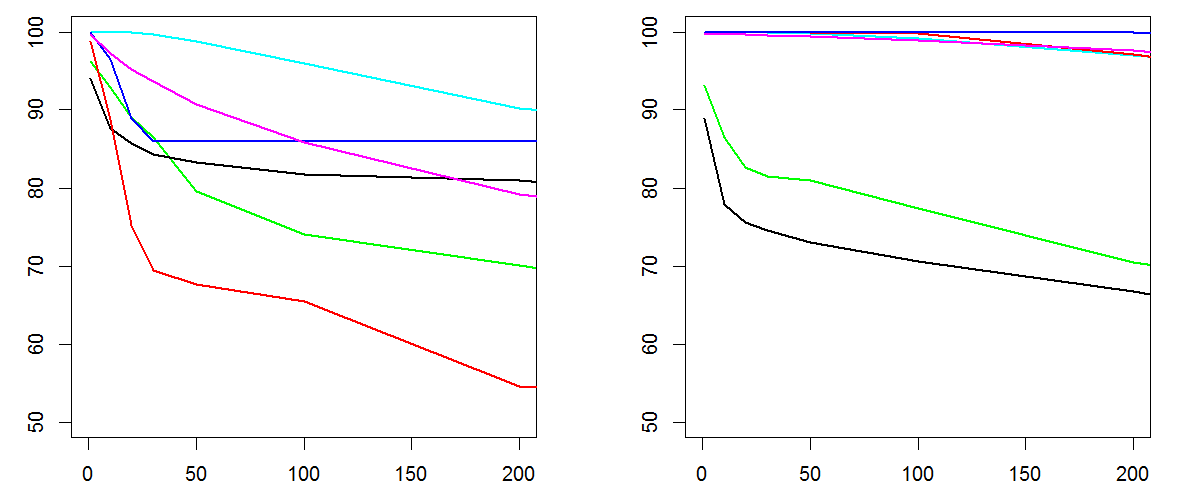
**

Here we show the percentage of each target gene that is covered at ≥ x sequencing depth, calculated as a mean across all samples.The lower panels show the same data, with a larger scale on the x-axis. On the PGM, two genes (KCNQ1 & KCHN2) show a sharp drop-off in coverage, suggesting that some regions are difficult to robustly sequence. On the MiSeq, KCNE1 and KCNE2 also showed significant drop-off.

**Figure S5. Sequencing coverage of target genes**

1. **MiSeq**

**
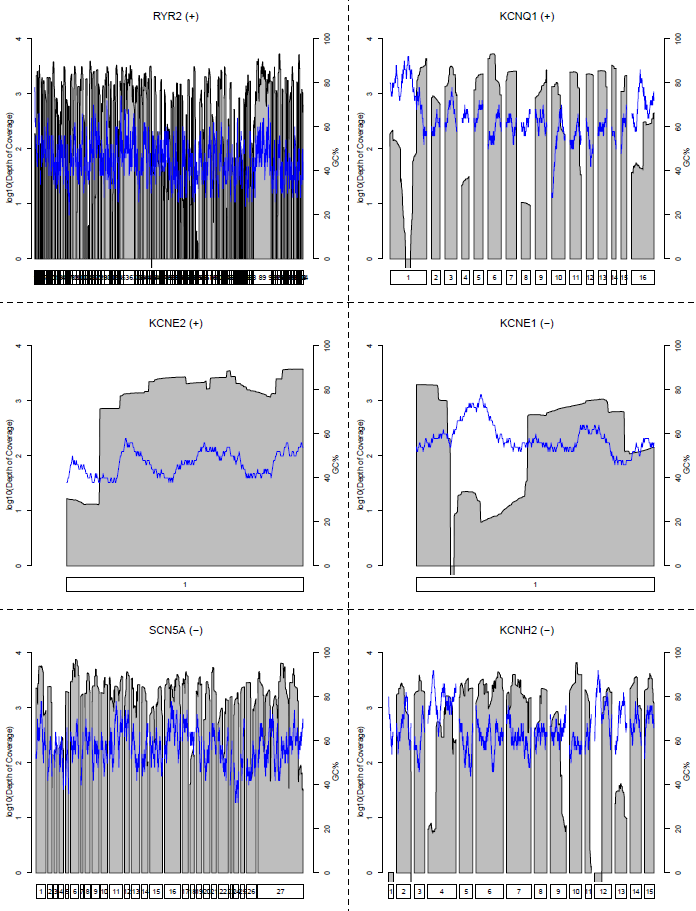
**

1. **Ion Torrent PGM**

**
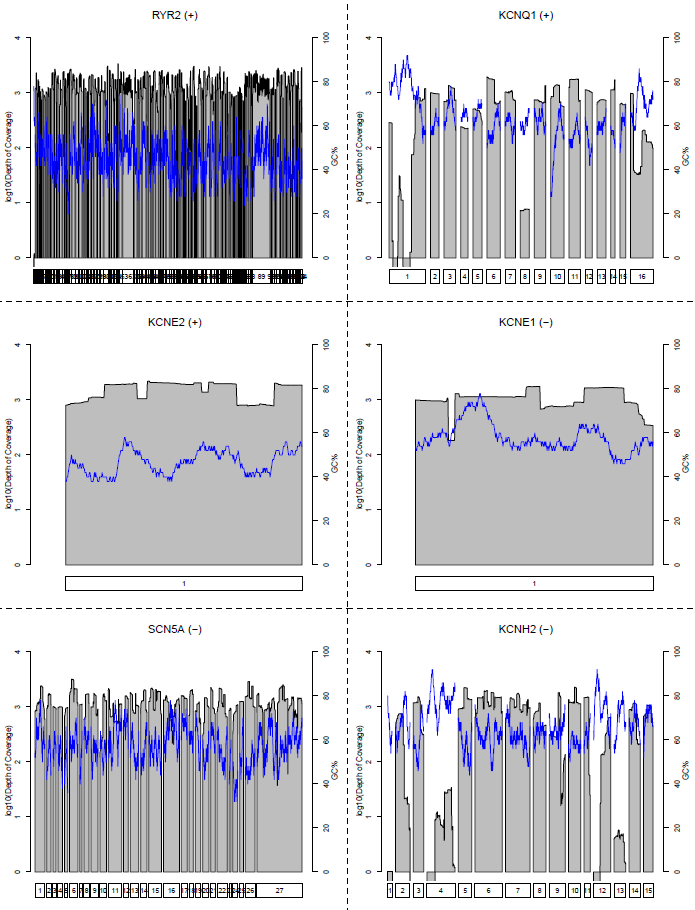
**

Sequencing depth is plotted for each coding base of the six target genes, on a log_10_ scale. Depth is calculated as a mean across 15 samples. Regions covered by a single read are therefore plotted at the origin, and regions of zero coverage have a negative deflection on the y-axis. GC content (calculated with a 50bp sliding window on the genomic DNA forward strand) is overlaid in blue. Plus (+) or minus (-) indicates the strand on which each gene is encoded.

While some regions are clearly problematic for both platforms (e.g. KCNQ1 exon 2, KCNH2 exons 1 & 12), there are also regions where one platform performs better (e.g. KCNE1, KCNE2, KCNH2 exon 4).

**Figure S6. The relationship between GC content and coverage.**

**
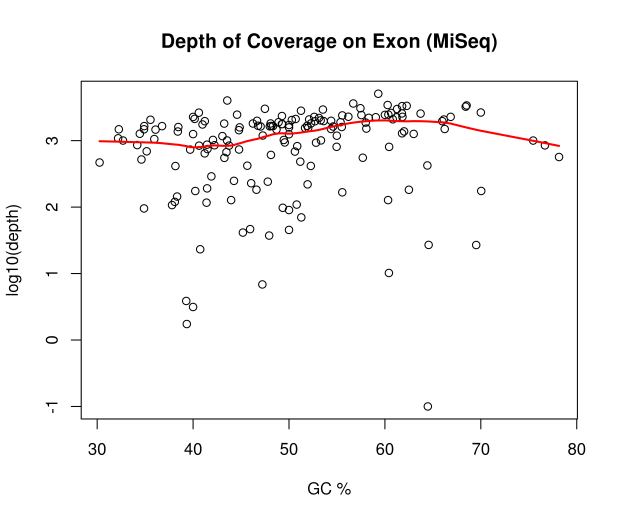

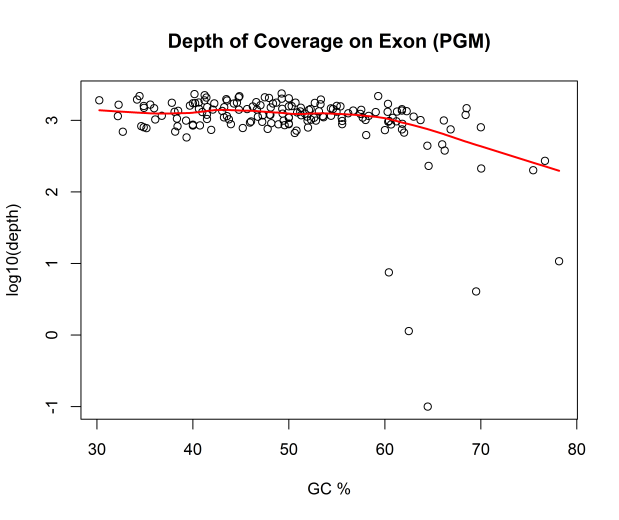
**

Sequencing depth (log_10_ scale) for each exon is plotted against its GC content. The coefficient of variation is larger for MiSeq than for Ion Torrent PGM (0.931 vs. 0.407). Loess regression is shown in red. MiSeq performance appears more variable across the GC range, whereas Ion Torrent performance falls off at high GC values, perhaps because of the additional emulsion PCR.

**Table S1. Barcode indexes and Ion Torrent specific adapters**

| **Primer** | **Orientation** | **Sequence (5' to 3')** |
| --- | --- | --- |
| A_BC6_CS1 | Sense | CCATCTCATCCCTGCGTGTCTCCGACTCAGACTGACTGACACACTGACGACATGGTTCTACA |
| A_BC7_CS1 | Sense | CCATCTCATCCCTGCGTGTCTCCGACTCAGAGATTGTAGCACACTGACGACATGGTTCTACA |
| A_BC8_CS1 | Sense | CCATCTCATCCCTGCGTGTCTCCGACTCAGCTCCTGTGGCACACTGACGACATGGTTCTACA |
| A_BC9_CS1 | Sense | CCATCTCATCCCTGCGTGTCTCCGACTCAGATATGCAACCACACTGACGACATGGTTCTACA |
| A_BC10_CS1 | Sense | CCATCTCATCCCTGCGTGTCTCCGACTCAGACAGACACGCACACTGACGACATGGTTCTACA |
| CS1_P1 | Anti-sense | CCTCTCTATGGGCAGTCGGTGATACACTGACGACATGGTTCTACA |
|  |  |  |
| A_BC6_CS2 | Sense | CCATCTCATCCCTGCGTGTCTCCGACTCAGACTGACTGACTACGGTAGCAGAGACTTGGTCT |
| A_BC7_CS2 | Sense | CCATCTCATCCCTGCGTGTCTCCGACTCAGAGATTGTAGCTACGGTAGCAGAGACTTGGTCT |
| A_BC8_CS2 | Sense | CCATCTCATCCCTGCGTGTCTCCGACTCAGCTCCTGTGGCTACGGTAGCAGAGACTTGGTCT |
| A_BC9_CS2 | Sense | CCATCTCATCCCTGCGTGTCTCCGACTCAGATATGCAACCTACGGTAGCAGAGACTTGGTCT |
| A_BC10_CS2 | Sense | CCATCTCATCCCTGCGTGTCTCCGACTCAGACAGACACGCTACGGTAGCAGAGACTTGGTCT |
| CS2_P1 | Anti-sense | CCTCTCTATGGGCAGTCGGTGATTACGGTAGCAGAGACTTGGTCT |

Primers used for Ion Torrent PGM barcoded library prep, with index sequences highlighted. Each amplicon is inserted into the complex in both orientations:

A-adaptor_Barcode_CommonSequence1_Amplicon_CommonSequence2_P1-adaptor

A-adaptor_Barcode_CommonSequence2_Amplicon_CommonSequence1_P1-adaptor

**Table S2. Detected variant information**

LRG = Locus Reference Genomic; Chr = Chromosome; Ref = reference allele; Alt = Alternative allele

P = variants discovered by PGM; M = variants discovered by Miseq; Highlighted SNP missed by both platforms

Note: All variants appearing in this table were confirmed by Sanger DNA sequencing analysis.
